# Cerebrospinal fluid penetration of fosfomycin in patients with ventriculitis: an observational study

**DOI:** 10.1186/s12941-023-00572-4

**Published:** 2023-04-24

**Authors:** Christina König, Jens Martens-Lobenhoffer, Patrick Czorlich, Manfred Westphal, Stefanie M. Bode-Böger, Stefan Kluge, Jörn Grensemann

**Affiliations:** 1 Department of Intensive Care Medicine, University Medical Center Hamburg-Eppendorf, Martinistraße 52, 20246 Hamburg, Germany; 2 Hospital Pharmacy, University Medical Center Hamburg-Eppendorf, Martinistraße 52, 20246 Hamburg, Germany; 3 Institute of Clinical Pharmacology, Otto-Von-Guericke University, Leipziger Str. 44, 39120 Magdeburg, Germany; 4 Department of Neurosurgery, University Medical Center Hamburg-Eppendorf, Martinistraße 52, 20246 Hamburg, Germany

**Keywords:** Critical Care (D003422), Drug Monitoring (D016903), Pharmacokinetics (D010599), Fosfomycin (D005578), Cerebral Ventriculitis (D058565)

## Abstract

**Background:**

For treatment of ventriculitis, vancomycin and meropenem are frequently used as empiric treatment but cerebrospinal fluid (CSF) penetration is highly variable and may result in subtherapeutic concentrations. Fosfomycin has been suggested for combination antibiotic therapy, but data are sparse, so far. Therefore, we studied CSF penetration of fosfomycin in ventriculitis.

**Methods:**

Adult patients receiving a continuous infusion of fosfomycin (1 g/h) for the treatment of ventriculitis were included. Routine therapeutic drug monitoring (TDM) of fosfomycin in serum and CSF was performed with subsequent dose adaptions. Demographic and routine laboratory data including serum and CSF concentrations for fosfomycin were collected. Antibiotic CSF penetration ratio as well as basic pharmacokinetic parameters were investigated.

**Results:**

Seventeen patients with 43 CSF/serum pairs were included. Median fosfomycin serum concentration was 200 [159–289] mg/L and the CSF concentration 99 [66–144] mg/L. Considering only the first measurements in each patient before a possible dose adaption, serum and CSF concentrations were 209 [163–438] mg/L and 104 [65–269] mg/L. Median CSF penetration was 46 [36–59]% resulting in 98% of CSF levels above the susceptibility breakpoint of 32 mg/L.

**Conclusion:**

Penetration of fosfomycin into the CSF is high, reliably leading to appropriate concentrations for the treatment of gram positive and negative bacteria. Moreover, continuous administration of fosfomycin appears to be a reasonable approach for antibiotic combination therapy in patients suffering from ventriculitis. Further studies are needed to evaluate the impact on outcome parameters.

**Supplementary Information:**

The online version contains supplementary material available at 10.1186/s12941-023-00572-4.

## Background

In acute hydrocephalus, external ventricular drains (EVD) are often used as temporary devices for the therapeutic diversion of cerebrospinal fluid (CSF). As a percutaneously placed catheter, EVDs are prone to bacterial colonization that can lead to ventriculitis, requiring antibiotic therapy [1]. As opposed to meningitis, little meningeal inflammatory exists in ventriculitis, resulting in only little antibiotic penetration into the CSF [2]. Therefore, despite high-dose antibiotic therapy subtherapeutic CSF levels may be present. To prevent from subtherapeutic concentrations a routine therapeutic drug monitoring (TDM) from the cerebrospinal fluid (CSF) has been established in our institution to adjust antibiotic therapy accordingly [3]. This is only possible within certain limits as toxicity may occur at high serum levels, mandating the measurement of serum concentrations, as well.

As initial empirical therapy, meropenem and vancomycin are indicated [4] but current data shows variable and often low penetration into the CSF [5]. Therefore, combination antibiotic therapy has been suggested to overcome this problem [4]. Fosfomycin exerts a broad-spectrum bactericidal activity covering both gram positive and negative bacteria including *Staphylococcus spp.* and *Pseudomonas spp.* As recently discussed [6] fosfomycin also shows biofilm activity against *Staphylococcus spp.* which makes it suitable for the treatment of device associated central nervous system (CNS) infections. Therefore, the local antibiotic therapy standard for EVD associated ventriculitis includes adding fosfomycin where timely removal or replacement of EVD is not possible. Fosfomycin is eliminated renally and shows a half-life of approximately 2 h in adults with normal renal function. With a small volume of distribution (V_d_), small molecular size and almost no protein-binding fosfomycin is distributing widely into all body compartments. Preliminary results have shown that fosfomycin penetrates well into the CSF even in the absence of meningeal inflammation [2, 7, 8]. Even though, current data show that fosfomycin exhibits time-dependent bactericidal activity it is most often used as intermittent infusion. Assuming that fosfomycin pharmacokinetics are relatively similar to meropenem, where continuous infusion lead to more sufficient CSF levels [3, 9], this administration type was adopted for fosfomycin. Moreover, this approach has been used previously in CNS infections with fosfomycin being administered by continuous infusion (24 g/24 h) [10, 11]. As this approach has not been systematically studied, we aimed to investigate fosfomycin CSF concentrations attained in neurocritical care patients with device (EVD) associated ventriculitis.

## Methods

### Study design and population

This analysis was reported to the Ethics Committee of the Hamburg Chamber of Physicians (Reference: WF-028/20, February 11, 2020). Due to the non-interventional nature of this study and anonymous recording of data, written informed consent was waived.

Patients included were diagnosed with ventriculitis and treated with fosfomycin by continuous infusion as combination therapy with meropenem and vancomycin as initial empiric therapy. TDM measurements were performed in regular intervals during EVD diagnostics from serum and CSF. Antibiotic dosages were adjusted according to TDM results from serum and CSF samples. Moreover, antibiotic regimens were deescalated, adjusted, or discontinued after species identification or at the discretion of the treating physician.

Diagnosis of ventriculitis was generally applied according to the CDC/NHSN surveillance definition [12]. Since clinical criteria like meningeal or cranial nerve signs could not be obtained in some patients due to sedation, impaired consciousness or interfering neurological deficits, a suspicion for ventriculitis and subsequent treatment indication was solely based on pathological CSF parameters such as increased leucocytes, elevated protein, decreased absolute glucose or decreased CSF/serum glucose ratio also if no growth was seen in the CSF culture in these cases.

### Data collection

Demographic and clinical data were obtained from the patients’ electronic records (Integrated Care Manager ICM, version 10.1, Drägerwerk AG, Lübeck, Germany, and Soarian Clinicals 4.01 SP08, Cerner Health Services, Idstein, Germany). We recorded data on antibiotic serum and CSF concentrations. Renal function was determined by the estimated glomerular filtration rate (eGFR) calculated according to the Chronic Kidney Disease Epidemiology Collaboration (CKD-EPI) [13]. Moreover, clinical routine data such as CSF parameters as well as microbiological results from CSF and blood cultures were obtained. As fosfomycin is available as a disodium salt, serum sodium levels were collected from the blood gas analysis (BGA), as well. The Simplified Acute Physiology Score II (SAPS II) [14] was recorded as a measure of disease severity. Outcome was assessed at discharge according to the Glasgow Outcome Scale (GOS), which consists of the five categories *death* (1), *persistent vegetative state* (2), *severe* (3), *moderate* (4) and *low disability* (5) [15].

### Drug administration

Fosfomycin (Infectopharm, Heppenheim, Germany) was reconstituted with water and used with a final concentration of 100 mg/mL. It was administered by continuous intravenous infusion (CI) with an initial dose of 1 g/h. Considering a potential disequilibrium effect in CSF two times the minimal inhibitory concentration (MIC) was targeted. Regarding the general European Committee on Antimicrobial Susceptibility Testing (EUCAST) breakpoint for susceptibility (32 mg/L) 64 mg/L was targeted. For *Pseudomonas spp.* and *Enterococcus spp.*, a target concentration above 128 mg/L was aimed for. This represented a pragmatic approach adapted from the published wild-type MIC distributions although neither an epidemiological cut-off value (ECOFF) nor a breakpoint has been defined in these cases by the EUCAST, yet [16]. Fosfomycin exposure in serum and CSF was optimized by adapting the infusion rate taking into account a penetration rate of approximately 30% [17].

### Bioanalytical method

Fosfomycin serum samples were collected and centrifuged (5000 rpm, 10 min, 20 °C), subsequently. The serum supernatant and CSF samples were stored at dry ice until being further processed. Serum samples were analyzed by LC–MS/MS according to the procedure described by Martens-Lobenhoffer et al. [18]. CSF samples were prepared and analyzed in a similar manner. Calibration ranges were 15–750 mg/L in serum as well as in CSF. Coefficients of variation for serum samples were 6.0% at 15 mg/L and 4.0% at 750 mg/L, respectively, with accuracies of 10.9% to − 0.2%. The corresponding coefficients of variation in CSF were 5.6–3.6%, with accuracies of − 13.9–9.7%. The lower limits of quantification were set to the lower end of the calibration range of 15 mg/L for both matrices. Samples with concentrations above the upper limit of quantification (750 mg/L) were diluted with blank serum (or water in case of CSF samples) and were re-analyzed.

### Statistics

Data management, non- compartmental calculations for fosfomycin clearance (CL) and area under the curve (AUC) were performed by using Microsoft Excel 365 (Microsoft Corp., Redmond, WA, USA). CL (Eq. 1) and AUC (Eq. 2) were calculated as follows:

1 $$CL \left[ {{L \mathord{\left/ {\vphantom {mg L}} \right. \kern-0pt} h}} \right] = \frac{{{\text{fosfomycin}}\,{\text{dose}}\,\left[ {{{mg} \mathord{\left/ {\vphantom {{mg} {24h}}} \right. \kern-0pt} {24h}}} \right]}}{{{\text{fosfomycin}}\,{\text{concentration}}\,{\text{in}}\,{\text{serum}}\,\left[ {{{\text{mg}} \mathord{\left/ {\vphantom {{\text{L}} {\text{L}}}} \right. \kern-0pt} {\text{L}}}} \right]\, * \,24\,{\text{h}}}}\,$$

2 $$AUC\left[ {{\raise0.7ex\hbox{${mg\!\,^*h}$} \!\mathord{\left/ {\vphantom {{mg} {L}}}\right.\kern-0pt} \!\lower0.7ex\hbox{${L}$}}} \right] = \frac{{{\text{fosfomycin}}\,{\text{dose}}\left[ {{{{\text{mg}}} \mathord{\left/ {\vphantom {{{\text{mg}}} {{\text{24h}}}}} \right. \kern-0pt} {{\text{24h}}}}} \right]}}{{{\text{CL}}\,\left[ {{{\text{L}} \mathord{\left/ {\vphantom {{\text{L}} {\text{h}}}} \right. \kern-0pt} {\text{h}}}} \right]}}$$

Visualization, statistical evaluation as well as determination of coefficients of correlation (R^2^) were either calculated by linear or non-linear regression methods included in Prism 9 (GraphPad Software, San Diego, CA, USA).

## Results

Between January 2020 and October 2021, 43 pairs of serum and CSF concentrations of fosfomycin were obtained from 17 patients treated for device associated ventriculitis. An overview on patient characteristics is given in Table 1.

**Table 1:** Patient characteristics

Age [years]	57 ± 12
Gender	Male: 59%
	Female: 41%
Body height [cm]	175 ± 7
Weight [kg]	84 ± 20
BMI [kg/m^2^]	27 ± 5
Creatinine [mg/dL]	0.64 ± 0.36
eGFR CKD-EPI [ml/min/1.73m^2^]	110 ± 32
Serum sodium [mmol/L]	142 ± 8
GOS	GOS 1: 2 (12%)
	GOS 2: 1 (6%)
	GOS 3: 11 (65%)
	GOS 4: 0 (0%)
	GOS 5: 3 (17%)
Underlying disease	Aneurysmal subarachnoid hemorrhage: 8 (47%)
	Neoplasia: 4 (23%)
	Intracerebral hemorrhage: 3 (18%)
	Ventricular-peritoneal shunt infection: 2 (12%)
LOS [days]	25 [21–52]
Inflammation markers on day 1 of fosfomycin treatment	
CSF glucose [mg/dL]	530 [442–650]
CSF cell count [× 10^6^/L]	582 [162–2562]
CSF protein level [mg/dL]	1163 [802- 2393]
CSF lactate [mmol/L]	5 ± 2
CSF IL-6 [pg/mL]	1782 [61–13229]
SAPS II	36 [29–60]
White blood count in serum [x 10^9^/L]	9.4 ± 5.4
C-reactive protein [mg/L]	67 [27–99]

Data are shown as the mean ± standard deviation or median with interquartile range [IQR]

*BMI* body mass index, *eGFR* estimated glomerular filtration rate, *CSF* cerebrospinal fluid, *CKD-EPI* chronic kidney disease epidemiology collaboration, *IL-6* interleukin 6, *SAPS II* simplified acute physiology score II, *GOS* glasgow outcome scale, *LOS* length of stay

The patients showed a regular body composition (body mass index 27 ± 5 kg/m^2^) and a preserved renal function with a mean estimated GFR of 110 ± 32 ml/min/1.73m^2^. Fosfomycin dosing was started with 24 g/24 h and adapted to a mean fosfomycin dosing regimen of 20 g/24 h during the course of the therapy.

**Fig. 1:**
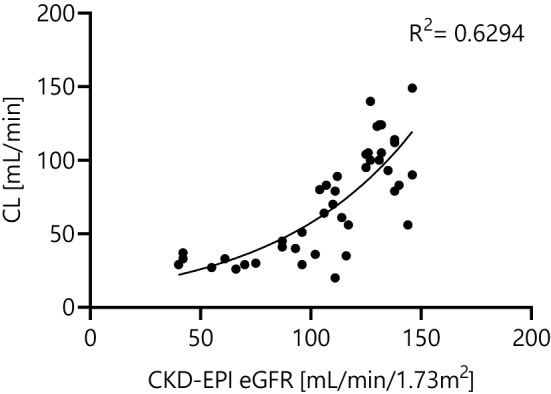
Fosfomycin clearance vs. CKD-EPI eGFR. *CKD-EPI* chronic kidney disease epidemiology equation, *CL* clearance, *eGFR* estimated glomerular filtration rate, R^2^- coefficient of correlation

The mean fosfomycin clearance was 4.2 ± 2.2 L/h and correlated best with the CKD-EPI eGFR values with a coefficient of correlation R^2^ = 0.63 (see Fig. 1).

The median attained serum concentration of fosfomycin was 200 [159–289] mg/L and the median CSF concentration 99 [66–144] mg/L (Table 2). When considering only the first measurements before a possible dose adaption, the median serum and CSF concentrations were 209 [163–438] mg/L and 104 [65–269] mg/L, respectively. The median area under the curve (AUC) was 4800 [3816–7152] in serum and 2381 [1585–3456] mg*h/L in CSF, respectively. The time course of fosfomycin serum and CSF levels are presented in supplement Fig. 1.

**Table 2:** Fosfomycin general pharmacokinetics

Fosfomycin [g/24 h]	20 ± 6
Fosfomycin therapy [d]	13 ± 9
Fosfomycin CL [L/h]	4.2 ± 2.2
Fosfomycin serum level [mg/L] first measurement	209 [163–438]
Fosfomycin CSF level [mg/L] first measurement	104 [65–269]
Fosfomycin serum level [mg/L]—all samples	200 [159–289]
Fosfomycin CSF level [mg/L]—all samples	99 [66–144]
Fosfomycin penetration rate [%]	46 [36–59]
Fosfomycin AUC in serum [mg*h/L]	4800 [3816–7152]
Fosfomycin AUC in CSF [mg*h/L]	2381 [1586–3456]
AUC/MIC (32 mg/L) ratio in serum	150 [119–224]
AUC/MIC (32 mg/L) ratio in CSF	74 [50–108]

Data are shown as the mean ± standard deviation or median with interquartile range [IQR]

*AUC* area under the curve, *CL* clearance, *CSF* cerebrospinal fluid, *MIC* minimal inhibitory concentration

**Fig. 2:**
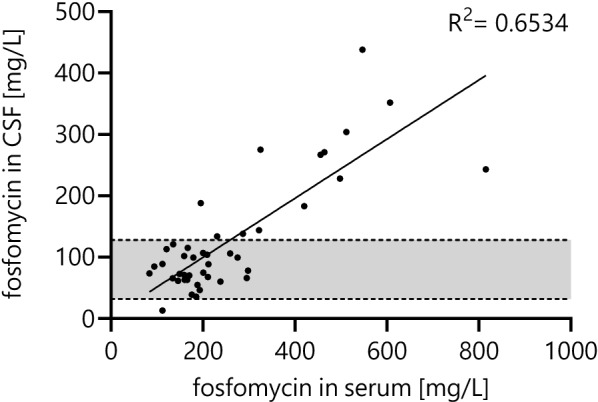
Fosfomycin concentrations in serum and cerebrospinal fluid. *CSF* cerebrospinal fluid, *MIC* minimal inhibitory concentration, light grey shaded areas depict concentrations within targeted range of 32 to 128 mg/L of fosfomycin

The CSF penetration for fosfomycin was 46 [36–59]% with an R^2^ of 0.65 (Fig. 2). Penetration ratio per GOS group is presented in supplement Table 1.

**Fig. 3:**
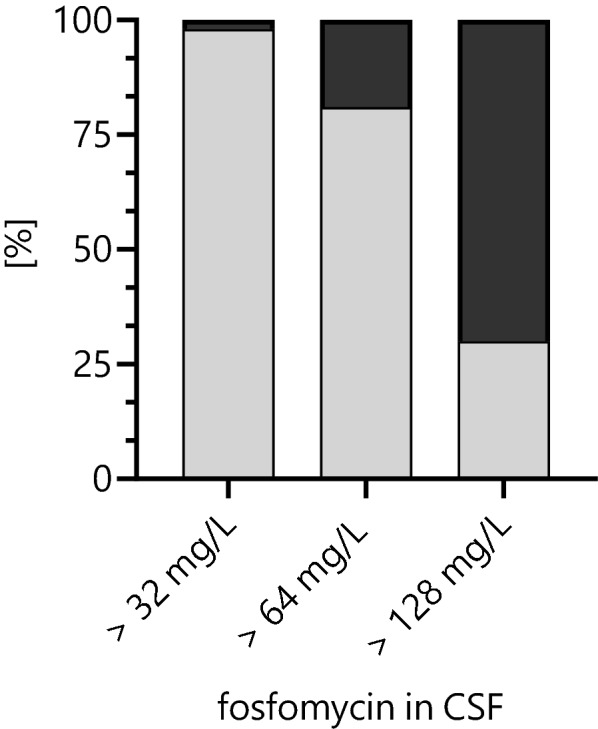
Percent fosfomycin concentrations in CSF above a given target. *CSF* cerebrospinal fluid. Black bars depict values not achieving a given threshold

All CSF values but one were above the EUCAST breakpoint of 32 mg/L (98%) and 13 CSF values (30%) were above 128 mg/L. In total, 98% of the observed CSF concentrations were above the MIC of 32 mg/L for susceptible isolates (see Fig. 3). When targeting *Pseudomonas* species with an ECOFF of 128 mg/L 30% of the analyzed CSF specimens achieved this concentration. The corresponding AUC/MIC ratio in CSF for an MIC of 32 mg/L was 74 [50–108].

Most patients received fosfomycin as a part of a combination therapy including meropenem and/or vancomycin. Two patients were deescalated to fosfomycin monotherapy due to infections with *Staphylococcus epidermidis.* Microbiological results are shown in Table 3.

**Table 3:** Microbiological results of CSF cultures ^a^

Pathogens	*Staphylococcus* *epidermidis* (n = 5)
	other coagulase negative *Staphylococci* (n = 5)
	*Staphylococcus aureus* (n = 1)
	*Enterobacter cloacae* (n = 4)
	*Klebsiella pneumoniae* (n = 1)
	*Citrobacter koseri* (n = 1)
	no pathogens isolated (n = 4)

^a^More than one pathogen was detected in four patients

Within the cohort, serum sodium concentrations ranged between 124 to 167 mmol/L with a mean value of 142 mmol/L. Hypernatremia was already present in 47% (n = 8) of the patients prior to fosfomycin treatment start with a mean sodium value of 153 mmol/L in this subgroup.

## Discussion

As opposed to meningitis, reaching sufficient bactericidal concentrations in ventriculitis may be difficult. Diffusion of anti-infectives into the CNS is dependent on different factors such as their lipophilicity, protein binding and molecular size [19]. Moreover, meningeal inflammation leads to the opening of tight junctions allowing for the penetration of drugs into the CSF whereas in ventriculitis meningeal inflammation is often not present [19]. Therefore, intermittent infusions might insufficiently attain appropriate plasma-CSF-equilibration resulting in lower penetration ratios. To date, no method allows for a quantification of meningeal inflammation and therefore approximations regarding drug CSF penetration ratios often remain uncertain. This problem was thought to be overcome by administering fosfomycin as a continuous infusion (CI), thereby permanently increasing the diffusion gradient towards the CSF accompanied by routine TDM of serum und CSF samples.

To the best of our knowledge, this is the first study analyzing the penetration whilst using fosfomycin by a CI regimen. Application of fosfomycin by CI or prolonged infusion has been studied by *in-vitro* and *in-vivo* analyses [20] and was performed successfully in case studies before [21–23]. This is also in concordance with *in-vitro* data showing pharmacodynamic indexes such as either time-dependent pharmacodynamics for species such as *Staphylococcus aureus* or *Enterococcus spec.* [24] or AUC/MIC dependency for *Pseudomonas aeruginosa o*r *Escherichia coli* [25–27]*.* With the chosen dosing regimen fosfomycin (d1 24 g/d) penetration into the CSF was reliably reaching CSF concentrations well above the defined EUCAST breakpoint of 32 mg/L for susceptible bacteria such as *Staphylococcus aureus*. The AUC/MIC ratio for an MIC of 32 mg/L was 74 for CSF specimens. This is in concordance with *in-vitro* data showing bacteriostasis at mean AUC/MIC ratios of 22.7 and a 1-log kill for 83.3 when targeting the group of *Enterobacterales* [25]. Unfortunately, MICs of the identified isolates were not reported by the local laboratory and therefore, no MIC guided dose adaption could be performed. In general, the achieved mean AUC of 4800 [3816–7152] mg*h/L in serum was higher than in a previous study reporting 4411 mg*h/L in healthy volunteers receiving a CI of 1 g/h [28].

Because our cohort included one patient with renal insufficiency (eGFR < 50 mL/min/1.73m^2^), increased AUC and serum concentrations (> 400 mg/L) using the initial standard fosfomycin dose were seen.

The penetration ratio of 46% in our cohort is within the range of 30–60% as reported earlier by Silica et al. [11] who used intermittent infusion regimens with up to 16 g/d in patients with meningitis. Pfausler et al. conducted lower CSF concentrations of 62 ± 32 mg/L when using 8 g fosfomycin 8 hourly in patients with ventriculitis [17]. This translated into a penetration ratio of approximately 25% when compared to serum. However, higher CSF concentrations as required for MIC > 64 mg/L which are often present in *Pseudomonales* or other non-fermenting bacteria could not be attained in all specimens of our cohort, even though 24 g/d by continuous infusion was used empirically.

More recently, CSF protein levels could be associated with an improved CSF penetration ratio of meropenem in patients with ventriculitis [3], whereas in our cohort no such association with variables such as GOS or other laboratory parameters could be identified for fosfomycin.

Fosfomycin CI was generally well tolerated as it was infused via a central line. In cases where increased serum sodium levels occurred during treatment, hypernatremia was already present prior to fosfomycin start. This is in concordance with Putensen et al. who also found a moderate incidence for hypernatremia of 14.3% [29]. Besides, none of the treated patients had to discontinue fosfomycin treatment due to adverse events.

Our study has certain limitations. We performed an analysis from clinically obtained samples that were collected at irregular time intervals from patients with ventriculitis presenting different underlying pathologies. However, our data therefore represents “real-world” conditions from a mixed cohort of patients. Moreover, there is currently no guideline regarding required antibiotic CSF concentrations for the treatment of ventriculitis. Thus, we suggest that concentrations one to two times the MIC of the targeted pathogen are sufficient as inflammation occurs within the CSF and the surrounding tissue. On the other hand, tissue concentrations obtained by microdialysis as reported for meropenem can exceed the CSF concentrations by a factor of three [30]. Hence, if targeting either CSF concentrations representing a multiple of the MIC or if lower targets could also be sufficient for successful treatment remains a matter of debate. To clarify, further studies are needed to understand the pathophysiology of ventriculitis and to determine the antibiotic targets to optimize patient outcome.

## Conclusion

Fosfomycin administration by continuous infusion is a feasible way in patients with ventriculitis resulting in appropriate drug concentrations in CSF. Our patient cohort showed a high fosfomycin CSF penetration rate of 46%, making it a potential treatment option for susceptible gram negative and positive pathogens. Future studies are needed to systematically investigate fosfomycin treatment in infections of the central nervous system regarding efficacy and patient outcome.

## Supplementary Information

**Additional file 1: Additional Figure and Table; Figure 1**: Fosfomycin concentration in serum and CSF over time, **Table 1**: Fosfomycin concentration in CSF and penetration ratio per GOS

## Data Availability

The dataset used and/or analyzed during the current study are available from the corresponding author on reasonable request.

## Abbreviations

AUC: Area under the curve
CRP: C-reactive protein
CSF: Cerebrospinal fluid
ECOFF: Epidemiological cut-off values
EUCAST: European Committee on Antimicrobial Susceptibility Testing
EVD: External ventricular drain
GFR: Glomerular filtration rate
GOS: Glasgow outcome scale
LC–MS/MS: Liquid chromatography-tandem mass spectrometry
MIC: Minimal inhibitory concentration
SAPS II: Simplified acute physiology score II
TDM: Therapeutic drug monitoring
