# Supplementary material for: Cerebrospinal fluid penetration of fosfomycin in patients with ventriculitis: an observational study
Source: Ann Clin Microbiol Antimicrob. 2023 Apr 24;22:29. doi: 10.1186/s12941-023-00572-4 (PMC10127017; doi:10.1186/s12941-023-00572-4)
Supplement: Supplementary file 1 — Additional file 1: Additional Figure and Table; Figure 1: Fosfomycin concentration in serum and CSF over time, Table 1: Fosfomycin concentration in CSF and penetration ratio per GOS [file 12941_2023_572_MOESM1_ESM.docx]

**Supplemental material**

**Cerebrospinal fluid penetration of fosfomycin in patients with ventriculitis – an observational study**

Christina König^1,2^, Jens Martens-Lobenhoffer^3^, Patrick Czorlich^4^, Manfred Westphal^4^, Stefanie M. Bode-Böger^3^, Stefan Kluge^1^, Jörn Grensemann^1^

^1^ Department of Intensive Care Medicine, University Medical Center Hamburg-Eppendorf, Martinistraße 52, 20246 Hamburg, Germany

^2^ Hospital Pharmacy, University Medical Center Hamburg-Eppendorf, Martinistraße 52, 20246 Hamburg, Germany

^3^ Institute of Clinical Pharmacology, Otto-von-Guericke University, Leipziger Str. 44, 39120 Magdeburg, Germany

^4^ Department of Neurosurgery, University Medical Center Hamburg-Eppendorf, Martinistraße 52, 20246 Hamburg, Germany

**Figure 1:** Fosfomycin concentrations in serum and cerebrospinal fluid over time

**Table 1: Fosfomycin concentration in CSF and penetrationratio per GOS measure**

Data is presented as median and range

| GOS | Fosfomycin in CSF [mg/L] | Penetration ratio [%] |
| --- | --- | --- |
| 1 | 104 [74- 106] | 40 [37- 49] |
| 2 | 101 [70- 438] | 88 [41- 96] |
| 3 | 88 [13- 352] | 44 [12- 85] |
| 4 | - | - |
| 5 | 107 [99- 115] | 53 [36- 68] |
